# Photoreceptors Are Involved in Antioxidant Effects of Melatonin Under High Light in *Arabidopsis*

**DOI:** 10.3390/antiox14040458

**Published:** 2025-04-12

**Authors:** Ivan Bychkov, Anastasia Doroshenko, Natalia Kudryakova, Victor Kusnetsov

**Affiliations:** K.A. Timiryazev Institute of Plant Physiology RAS, 35 Botanicheskaya St., 127276 Moscow, Russia; ivan.a.b@mail.ru (I.B.); anastasiya04101993@gmail.com (A.D.); vkusnetsov2001@mail.ru (V.K.)

**Keywords:** *Arabidopsis thaliana*, high light stress, melatonin, mutants, reactive oxygen species

## Abstract

The beneficial role of melatonin (MT) as a potent broad-spectrum antioxidant and hormone-like regulator in plant protection against adverse environmental conditions is indisputable. However, the molecular networks underlying its unique scavenging capabilities are still far from understood. Herein, we show the ability of MT to maintain physiological functions under high light stress (HL) is mediated by photoreceptors. Melatonin treatment (50 μM) of the photoreceptor mutants *phyA/B* and *cry1/2* augmented the deleterious effects of excess light (600 μmol m^−2^ s^−1^, 24 h), as evidenced by increased TBARs levels and electrolyte leakage, as well as decreased photosynthetic efficiency, in contrast to their parental form, *Landsberg erecta*, in which these parameters were significantly improved. The reduced stress resistance of the mutants was also confirmed by analysis of the transcript accumulation of ROS markers and enzymatic scavengers. Moreover, the increase in melatonin content in the mutants exposed to HL + MT contributed to increased ROS accumulation; therefore, the deleterious effect of MT could not be explained by an imbalance in ROS production below the cytostatic level. We hypothesize that the light-sensitive phenotypes of photoreceptor mutants under MT treatment may be due to the misregulation of stress-related genes that are targets for melatonin action.

## 1. Introduction

Exposure of a plant to light levels exceeding those used in photochemistry results in the inactivation of photosynthetic functions and the formation of reactive oxygen species (ROS) [1]. In an attempt to decrease the production of ROS caused by exposure to excess light, plants have evolved multiple antioxidative mechanisms, including enzymatic and nonenzymatic scavengers. Enzymatic scavengers such as superoxide dismutase, ascorbate peroxidase (APX), glutathione reductase, monodehydroascorbate reductase, dehydroascorbate reductase (DHAR), and catalase can dismutate O_2_^−^ radicals and scavenge H_2_O_2_. Nonenzymatic antioxidants, including glutathione, ascorbate, tocopherol, and carotenoids, are engaged in ROS scavenging and the dissipation of excess light, thus preventing lipid oxidation [2].

Among nonenzymatic scavengers, melatonin (N-acetyl-5-methoxytryptamine) is of particular interest. Melatonin (MT) is a potent broad-spectrum antioxidant [3] capable of forming an antioxidant cascade, as a result of which one melatonin molecule can neutralize up to ten molecules of ROS [3]. However, the protective function of melatonin in plants is not limited to its direct antioxidant role. Melatonin, as a regulator, is able to modulate the activity of multiple genes associated with the plant response to stresses [4,5]. The dual role of melatonin as an antioxidant and a hormone-like regulator has been recognized in numerous studies, and it has been suggested that MT acts primarily as an antioxidant at high concentrations and as a regulator at low concentrations [6,7,8]. However, both functions often occur simultaneously.

Melatonin synthesis is induced under HL stress in *Arabidopsis*, which confers tolerance to high light stress (HL) either by scavenging ROS or by inducing various antioxidant enzymes [9]. Furthermore, exogenous melatonin treatment mitigates the damage caused by HL stress in the *snat1* knockout mutant line, which produces less melatonin than the wild type does by reducing O_2_^−^ production and increasing the expression of various ROS-responsive genes. In addition, melatonin has been shown to increase the photosynthetic rate and protect photosynthetic proteins under high light, particularly D1, D2, CP43, PsbS, LHCB1, LHCB2, LHCB3, LHCB4, LHCB, LHCB6, PSI-D, LHCA1, LHCA2, and LHCA3 [10]. This occurred because exogenous melatonin effectively decreased the accumulation of ROS and protected the integrity of the membrane and photosynthetic pigments. On the other hand, wild-type (WT) plants exposed to prolonged HL stress (600 µmol m^−2^ s^−1^ for 24 h) presented a two-fold decrease in melatonin content and downregulated melatonin biosynthetic genes (*SNAT1*, *ASMT*, and *COMT)*, which were partly restored following combined HL + MT treatment. In addition HL caused a decrease in the transcript accumulation of the nuclear-encoded photosynthetic gene *LHCB2* (*light-harvesting antenna protein of PSII*) and a set of selected chloroplast encoded genes transcribed by PEP *(rbcL*, *psbA*, *psbD*, *psaA*, and *trnE*), NEP (*accD*) or both polymerases (*atpB*), whereas melatonin maintained the expression of these genes at a higher level in the stressed plants [11]. Therefore, the pathways that regulate and coordinate plant responses to melatonin are diversified and specified by stress levels. This finding is consistent with the concept that stressors can produce different dose–response relationships depending on the specificity of the response and the ability or inability to overcome a particular stressor [12]. Notably, *Arabidopsis* mutant lines with disrupted genes for the melatonin signaling genes CAND2/PMTR1 and *GPA1*, which encode the MT receptor, and the α-subunit of the heterotrimeric G-protein were partially insensitive to melatonin treatment under high light stress. These compounds present a greater degree of photodamage, suggesting that under excessive light, melatonin may act as a hormone-like signaling molecule via the CAND2/PMTR1-mediated signaling pathway [11].

Plants perceive light signals through several groups of specific photoreceptors, among which phytochromes and cryptochromes play a key role in plant acclimation to high irradiance and increasing their survival [13,14]. A study of the high irradiance response of *Arabidopsis* photoreceptor mutants revealed that 77 of the high light-responsive genes are regulated via CRY1. As a consequence of the misregulation of these genes, the *cry1* mutant displayed a high irradiance-sensitive phenotype with significant photoinactivation of photosystem II, as indicated by a reduced maximal fluorescence ratio [14]. The CRY1-dependent response to excess light is mediated by the transcriptional activators ZML1 (ZIM-like 1) and ZML2 (ZIM-LIKE 2) in *Arabidopsis*. The *zml1* and *zml2* T-DNA insertion lines displayed severe photobleaching and misregulation of several CRY1-dependent genes (*ELIP2*, *GPX7*, *ERD9*, and *MYB7*) in response to high irradiance [15]. Recent studies have shown that the blue light cryptochrome photoreceptors UVR8 (regulator of chromosome condensation (RCC1) family protein) and phytochrome red photoreceptors converge on the induction of FAH1 (ferulic acid 5-hydroxylase 1), which encodes a key enzyme in the phenylpropanoid biosynthetic pathway, leading to the accumulation of UV-absorbing synaptic esters in *Arabidopsis* and providing photoprotection [16].

Phytochrome B (PhyB) is especially required for the plant response to HL stress. Fichman and colleagues undertook a detailed investigation of the role of PhyB in the plant response to different biotic and abiotic stresses [17]. The authors reported that PhyB is required for apoplastic ROS accumulation and functions as part of a signaling module with respiratory burst oxidase D (RBOHD) and F (RBOHF) coregulating thousands of genes during excess light stress. Surprisingly, in the absence of PhyB, ROS do not accumulate in plant cells under light stress, although *phyB* mutants display decreased acclimation to excess light. This finding is consistent with the concept that ROS play a dual role in plant biology and that maintaining an optimal range of ROS levels in cells is beneficial for plants. Many antioxidative systems maintain ROS at basal nontoxic levels, which are essential for life and are above the cytostatic level but below the cytotoxic level [18]. However, it is unknown whether melatonin is able to maintain the necessary balance of ROS under high irradiance, considering the role of photoreceptors in the regulation of ROS in response to excess light. Therefore, the aim of this study was to check whether photoreceptors are involved in the melatonin-mediated response to excess light. To test this issue, we subjected the double mutants *cry1/2* and *phyA/B* to long-term HL stress in the presence and absence of melatonin. Notably, we found that melatonin treatment of the photoreceptor mutants *phyA/B* and *cry1/2* failed to induce plant acclimation to HL stress and, unlike WT plants, exacerbated the deleterious effects of excessive light. We suggest that the ability of the regulation of MT-related genes to prevent ROS toxicity is mediated by photoreceptors.

## 2. Materials and Methods

### 2.1. Plant Material, Growth Conditions, and Treatments

*The A. thaliana* lines used in this study were in the *Landsberg erecta* (Ler) background. Seeds of the *cry1/2 double mutant (hy4-2.23Nfha-1)* were kindly provided by Professor Chentao Lin (University of California, Los Angeles, CA, USA), whereas the *phyA/B* mutant (*phyA-201phyB-5*) (NASC 6224) was obtained from the Nottingham Arabidopsis Stock Centre. Both mutants were verified via PCR genotyping. Seeds were stratified and germinated in half-strength Murashige and Skoog (MS) media supplemented with 1% sucrose and 0.5% agar under a 16 h light/8 h dark cycle at 23 °C and 60 μmol m^−2^ s^−1^ light intensity. Two-week-old plants were pretreated with 50 μM melatonin for 72 h and exposed for 24 h under an HPI-T2 2000 W/646 lamp (Philips, Amsterdam, The Netherlands) with a luminous energy flux of 600 μmol m^−2^ s^−1^. The control plants were kept under growing conditions. At the end of the experiment, the samples were immediately frozen in liquid nitrogen and refrigerated at −80 °C until use.

### 2.2. Determination of Chlorophyll and TBARs Contents and Electrolyte Leakage

Chlorophyll and carotenoids were extracted in a solution of 80% acetone, and their concentrations were determined colorimetrically at 665, 649, and 440 nm with a Pharmacia Biotech ultrospec 2000 spectrophotometer (London, UK) as described by Lichtenthaler [19].

The concentration of TBARs was assessed via a reaction with thiobarbituric acid according to Heath and Packer [20]. The absorbance of the extract was measured at wavelengths of 532 and 600 nm. Concentration calculations were performed via the following formula: C = D/EL, where C is the concentration of TBARs (μmol), D is the optical density, and E is the molar extinction coefficient equal to 1.56 × 10^5^ cm^−1^ M^−1^. The results are expressed in μmol/g of fresh weight.

Electrolyte leakage was measured with a Seven2Go S3 instrument equipped with an InLab 731-ISM electrode (Mettler Toledo, Hong Kong, China) and calculated as the percentage of the conductivity before autoclaving over that after autoclaving [21].

### 2.3. Quantification of Antioxidant Capacity, H_2_O_2_ and O_2_^−^

The total antioxidant activity was evaluated via a DPPH assay adapted from Brand-Williams et al. [22]. The plant material was homogenized at 2–4 °C in 80% ethyl alcohol solution and centrifuged for 10 min at 8000× *g* at 4 °C. The reaction was initiated by adding 2 mL of 200 μM DPPH solution to 1 mL of the extract. The reaction mixture was incubated for 30 min at room temperature in the dark. The absorbance was measured at 517 nm.

The H_2_O_2_ concentration was determined via a hydrogen peroxide assay kit (Sigma–Aldrich, St. Louis, MO, USA) according to the manufacturer’s recommendations. Plant tissue (100 mg) was homogenized in 200 μL of chilled 100% acetone and centrifuged for 10 min at 10,000× *g*. A total of 160 μL of the supernatant was transferred to clean tubes and mixed with 40 μL of 20% TiCl_4_ solution, followed by 40 μL of concentrated NH_4_OH solution. The precipitated titanium peroxide was washed with 100% acetone and resuspended in 2 N H_2_SO_4_ solution. The absorbance was measured at 415 nm, and the H_2_O_2_ content was calculated via a calibration curve of H_2_O_2_.

The rate of O_2_^−^ generation was assessed as follows [23,24]: The plant material was homogenized in 50 mM phosphate-buffer solution (pH 7.8) and centrifuged at 5000× *g*. The supernatant was mixed with extraction buffer and 10 mM hydroxylamine hydrochloride and incubated at 25 °C for 20 min. Then, 17 mM sulfanilamide and 7 mM naphthylamine were added, and the mixture was incubated again at 25 °C for 20 min. The optical density of the solution was measured at 530 nm. The rate of O_2_^−^ generation was calculated via the standard curve of NaNO_2_ [22,23].

### 2.4. Determination of Melatonin Content

The concentration of endogenous melatonin was quantified as outlined by Lee and Beck [9] via the CEA908GE ELISA Kit (Cloud-Clone Corp., Katy, TX, USA) according to the manufacturer’s protocol. Leaves (0.2 g) were ground in 2 mL of 100% chloroform and centrifuged for 15 min at 12,000× *g*. The supernatant was transferred into new tubes and placed in a vacuum evaporator until the liquid was completely removed. The precipitate was then dissolved in distilled water and vortexed for three hours. The optical density of the solution was measured at 450 nm via a Multiskan MS Microplate Reader LabSystem 352 (Thermo/LabSystems, Philadelphia, PA, USA).

### 2.5. Fluorescence Measurements

Chlorophyll fluorescence parameters (variable fluorescence to maximum fluorescence (Fv/Fm), the effective quantum yield of PSII (Φ_PSII_) and non-photochemical quenching NPQ) were measured via a DUAL-PAM101 (Walz, Effeltrich, Germany) as described by Kozuleva et al. [25]. The following parameters were determined: measuring light—460 nm; 9 µmol m^−2^ s^−1^, saturating pulses—500 ms, 635 nm, and 4000 µmol m^−2^ s^−1^; and actinic light—635 nm, 37 µmol m^−2^ s^−1^. The dark incubation time for the measurements was 30 min. Three to six leaves from three or four plants were used.

### 2.6. qRT–PCR Analysis

Total RNA was extracted from frozen leaves via the TRIzol (Thermo Fisher Scientific, Waltham, MA, USA) method. cDNA synthesis was performed from 2 μg of total RNA via a mixture of oligo (dT) primers and random hexamers. The primers (Table S1) used for real-time PCR analysis were designed via the Vector NTI 9 program. Quantitative real-time PCR was performed in a LightCycler 96 (Roche, Basel, Switzerland) with hot start SYBR Green I technology. The standard thermal profile for all PCRs included the following steps: 95 °C for 10 min; 40 cycles at 95 °C for 15 s, at 58 °C for 15 s, and at 72 °C for 20 s; and melting curve analysis. The relative transcript abundance of the tested genes was calculated via the 2^−ΔΔCt^ method and normalized to the expression level of *UBQ10.*

### 2.7. Statistical Analysis

At least three independent replicates were performed for the experiments. The significance of differences was estimated via one-way analysis of variance (ANOVA) followed by Tukey’s method via an online calculator (https://astatsa.com/OneWay_Anova_with_TukeyHSD/ (accessed on 11.04.2025)) and Student’s test. Significant differences are designated by different letters.

## 3. Results

### 3.1. Increased Melatonin Levels in Photoreceptor Mutants Alter Their Response to Melatonin Treatment Under HL Stress

To determine whether photoreceptors are involved in melatonin-mediated acclimation to excess light, we subjected 2-week-old WT plants to photooxidative stress (600 μmol m^−2^ s^−1^) for 24 h following preincubation for 3 days in a liquid 0.5 MS medium with or without the addition of 50 μM melatonin. The control plants were maintained at a light intensity of 60 μmol m^−2^ s^−1^. The concentrations of MT and the light intensity and duration were selected in preliminary experiments. Moderate HL stress conditions, which mimic realistic environmental stress conditions, do not cause apparent cell death but contribute to significant photodamage due to decreased photosynthetic activity and diminished expression of chloroplast and nuclear-encoded genes and the corresponding proteins [9,11].

**Figure 1:**
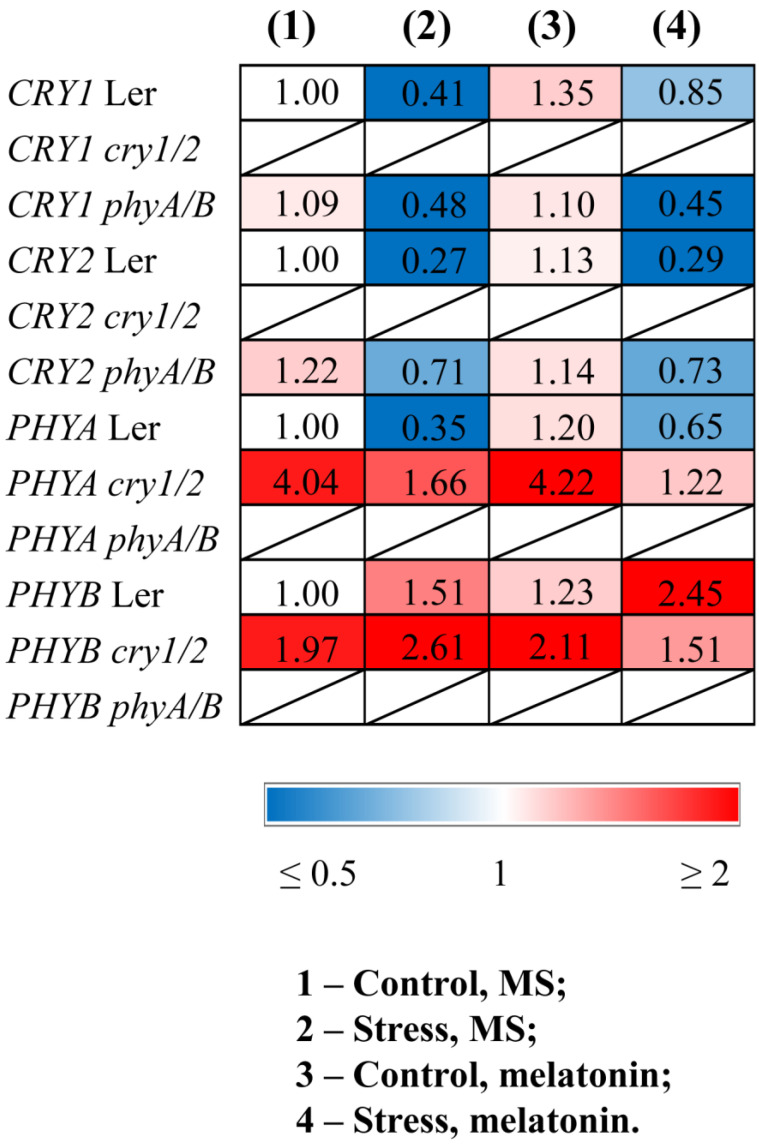
Effects of HL stress and melatonin on the expression of photoreceptor genes. Wild-type and mutant plants were grown on Murashige and Skoog media in Petri dishes for two weeks under a photoperiod of 16 h light/8 h dark at 23 °C and 60 μmol m^−2^ s^−1^ (control). Melatonin at a concentration of 50 μm was used for treatment. The experimental plants were exposed to light stress for 24 h at 600 μmol m^−2^ s^−1^ (stress). The numbers indicate the baseline ratio of the expression of each gene in the wild type and mutants without treatments and their values under experimental conditions. Statistical analysis for this figure is presented in Table S2.

Quantitative RT–PCR (qPCR) analysis revealed that stress decreased the transcript abundance of *CRY1*, *CRY2*, and *PHYA* and increased the expression of *PHYB* in WT plants (Figure 1, Table S2). Melatonin treatment of stressed WT plants partly restored the expression of *PHYA* and *CRY1* and contributed to further upregulation of *PHYB*. *PHYB* was also significantly activated by MT treatment in the control WT plants, confirming the role of melatonin in the transcriptional regulation of this gene. However, *PHYB* and *PHYA* were not responsive to MT treatment in the *cry1/2* mutant, and conversely, *CRY1* and *CRY2* did not respond to exogenous MT in the *phyA/B* mutant.

To examine the physiological role that melatonin plays in the response of photoreceptor mutants to HL stress, we measured the transcript levels of melatonin synthesis genes. Melatonin synthesis involves several steps, including the conversion of tryptophan to tryptamine by tryptophan decarboxylase (TDC), followed by the conversion of tryptamine into serotonin by tryptamine 5-hydroxylase (T5H). In the last steps, three enzymes are involved in the synthesis, including serotonin *N*-acetyltransferase (SNAT), *N*-acetylserotonin methyltransferase (ASMT), and caffeic acid *O*-methyltransferase (COMT) [26].

**Figure 2:**
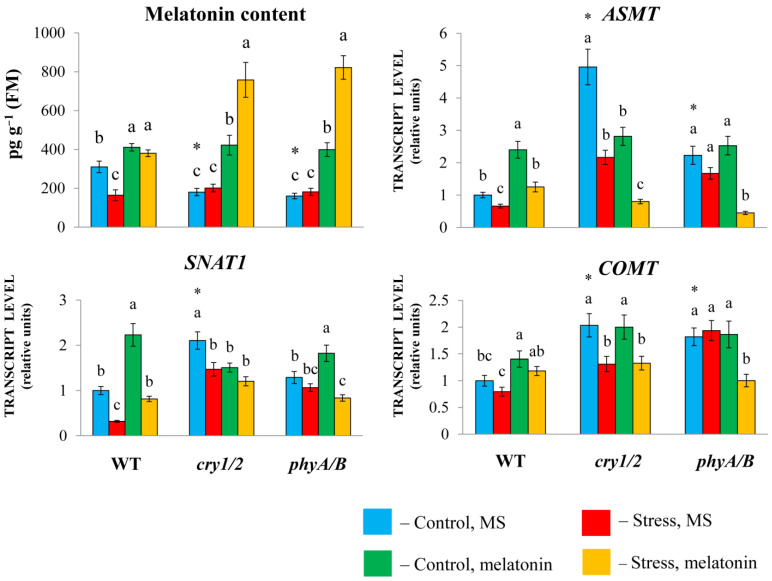
Effects of HL stress and exogenous melatonin on the endogenous melatonin content and the expression of melatonin synthesis genes wild-type and mutant plants were grown on Murashige and Skoog media in Petri dishes for two weeks under a photoperiod of 16 h in the light/8 h in the dark at 23 °C and 60 μmol m^−2^ s^−1^ (control). Melatonin at a concentration of 50 μm was used for treatment. The experimental plants were exposed to light stress for 24 h at 600 μmol m^−2^ s^−1^ (stress). The data presented in the figure are mean values (*n* ≥ 3). Error bars represent measurement errors. Different letters indicate statistically significant differences between variants within the same genotype at *p* < 0.05 (ANOVA with Tukey’s post hoc multiple comparison test). Asterisks indicate statistically significant differences between the mutants and the wild type under the corresponding treatment type at *p* < 0.05 (Student’s *t* test).

The expression of *SNAT1*, *COMT*, and especially *ASMT* was greater in both *cry1/2* and *phyA/B* than in their parental ecotype *Landsberg erecta* (LER). However, in *cry1/2* and *phyA/B* plants, the melatonin content was reduced (approximately two-fold) compared with that in wild-type plants, despite increased basic transcript levels of MT synthesis and signaling genes, likely due to compensatory feedback regulation (Figure 2).

Exogenous melatonin substantially increased the MT content in the mutants (more than two-fold), which further increased following the combined exposure to melatonin and HL. In wild-type plants, the melatonin content increased by only approximately 25% as a result of melatonin treatment, regardless of whether they were exposed to normal or excess light or combined stress and melatonin treatment.

Exogenous melatonin under moderate light increased the expression of MT synthesis genes in LER but did not substantially change their levels in the mutants or even reduce them compared with their elevated initial values. Excess irradiation resulted in a moderate decrease in the transcript abundance of MT genes in both the WT and the mutants. Moreover, compared with HL, the combined application of melatonin + HL caused an increase in the transcript abundance of MT synthesis genes in WT plants but did not significantly affect their levels in the mutants or even suppress them. These observations clearly indicate that increased melatonin levels lead to decreased expression of MT biosynthesis genes via negative feedback regulation. In summary, these results imply that the modes of action of exogenous melatonin may differ depending on the genetic background and initial MT content of the tested samples.

### 3.2. Increased Susceptibility to HL Stress in the Presence of MT in Photoreceptor Mutants Is Accompanied by Perturbed Physiological Indicators and Molecular Markers

**Figure 3:**
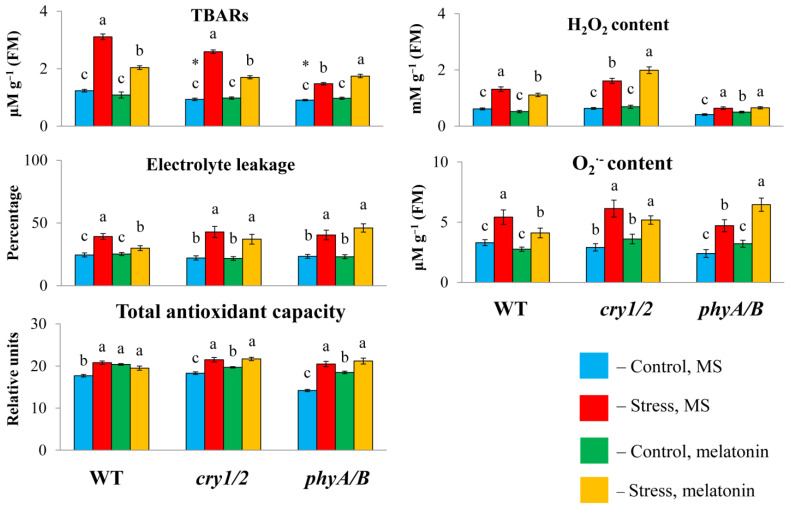
Effects of HL stress and melatonin on the TBAR content, H_2_O_2_ content, and electrolyte yield. Wild-type and mutant plants were grown on Murashige and Skoog media in Petri dishes for two weeks under a photoperiod of 16 h in the light/8 h in the dark at 23 °C and 60 μmol m^−2^ s^−1^ (control). Melatonin at a concentration of 50 μm was used for treatment. The experimental plants were exposed to light stress for 24 h at 600 μmol m^−2^ s^−1^ (stress). The data presented in the figure are mean values (*n* ≥ 3). Error bars represent measurement errors. Different letters indicate statistically significant differences between variants within the same genotype at *p* < 0.05 (ANOVA with Tukey’s post hoc multiple comparison test). Asterisks indicate statistically significant differences between the mutants and the wild type under the corresponding treatment type at *p* < 0.05 (Student’s *t* test).

To determine whether the failure of exogenous melatonin to induce MT synthesis genes under high irradiance could be associated with altered susceptibility to stress in photoreceptor mutants, we measured the TBAR content and electrolyte leakage, as these parameters are considered important indicators of oxidative damage. Under HL, the TBAR values were significantly greater in the WT and *cry1/2* plants than in the control plants and decreased with the simultaneous application of HL and melatonin (Figure 3). However, in the *phyA/B* mutant, the TBAR content, which increased by no more than 50% after HL stress, increased further under combined exposure to HL and melatonin instead of being reduced. Furthermore, the measurement of electrolyte leakage indicated that stress tolerance in both mutants was not altered following MT application, in contrast to WT plants, in which electrolyte leakage tended to decrease. Therefore, exogenous melatonin could not mitigate the photodamage to the cell membranes of the *phyA/B* and *cry1/2* mutants caused by excess light but alleviated the damage to the cell membranes in the LER.

We then analyzed changes in the total antioxidant capacity and ROS levels (hydrogen peroxide and O_2_^−^) since disruption of photoreceptor genes can perturb the balance between ROS production and scavenging. The total antioxidant capacity was significantly lower in *cry1/2* than in WT or *phyA/B* under control conditions. However, after treatment with HL or HL + MT, the values of the total antioxidant capacity increased and became almost the same in all the samples, indicating equal levels of antioxidant compounds. In parallel, exposure of the WT and the mutants to excess light resulted in the accumulation of ROS, which decreased in the WT following melatonin treatment but remained elevated in *cry1/2.* In *phyA/B*, the control levels of hydrogen peroxide and O_2_^−^ were substantially lower (by 1.5-fold) than those in the WT, and they increased after 24 h of HL.

However, melatonin treatment under stress conditions did not affect the level of ROS, which retained the values found in stressed plants (hydrogen peroxide) or even surpassed them (O_2_^−^). Furthermore, melatonin treatment of *phyA/B* under normal light resulted in a significant increase in the content of hydrogen peroxide and a substantial, albeit insignificant, increase in O_2_^−^, which is consistent with the assumption that MT increased the prooxidant capacity of the mutant. These results suggest that genetic defects in photoreceptor genes can abolish the ability of MT to scavenge ROS, enhancing oxidative damage.

**Figure 4:**
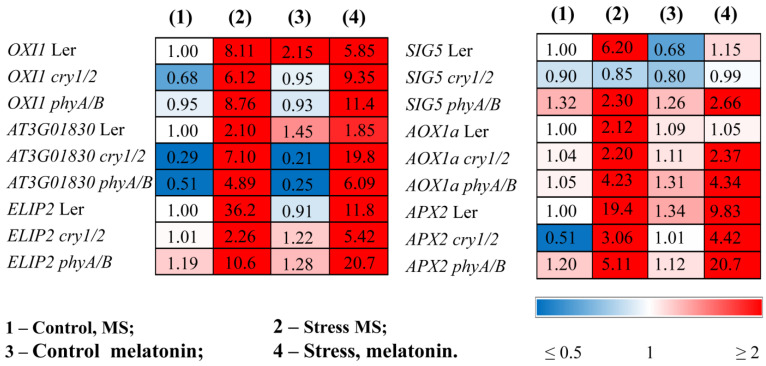
Effects of HL stress and melatonin on the expression of stress marker genes. Wild-type and mutant plants were grown on Murashige and Skoog media in Petri dishes for two weeks under a photoperiod of 16 h in the light/8 h in the dark at 23 °C and 60 μmol m^−2^ s^−1^ (control). Melatonin at a concentration of 50 μm was used for treatment. The experimental plants were exposed to light stress for 24 h at 600 μmol m^−2^ s^−1^ (stress). The numbers indicate the baseline ratio of the expression of each gene in the wild type and mutants without treatments and their values under experimental conditions. Statistical analysis for this figure is presented in Table S2.

The finding that the mutants are more susceptible to HL stress in the presence of melatonin was confirmed by ROS marker analysis and transcript accumulation of the key components of the photosynthetic light stress response. The expression of *OXI1* (the OXIDATIVE STRESS INDUCIBLE 1 kinase), a generic oxidative stress marker, and *At3g01830*, which is specifically induced by singlet oxygen [27], was significantly greater in *phyA/B* (2- and 3-fold, respectively) following the mutual application of MT and HL than the elevated levels induced by HL alone (Figure 4). Similar results were obtained for *cry1/2.* In contrast, melatonin contributed to their decrease in LER subjected to HL (Figure 4, Table S2).

*ELIP2* (*early light-inducible protein 2*), which is used as an indicator of light stress and encodes a protein with photoprotective functions [28], followed that of *OXI1*, and in the mutants presented the highest levels after the combined application of MT and HL. The stress-induced expression of *SIG5*, encoding a transcriptional sigma factor that is essential for the activity of plastid-encoded RNA polymerase in *Arabidopsis* chloroplasts, and *AOX1a*, encoding an alternative oxidase enzyme, had similar expression values regardless of whether the plants were treated with HL or melatonin + HL (Figure 4).

The efficiency of photoprotection can also be assessed by the activity of the enzymes directly involved in scavenging mechanisms and the expression of their corresponding genes. Light treatment caused an increase in the transcript abundance of *APX2*, encoding ascorbate peroxidase, in both the WT and mutants, although the range of increase varied, with mutants exhibiting reduced responsiveness (3- and 5-fold in *cry1/2* and phyA/B vs. 20-fold in the WT). The lower levels of *APX2* transcripts in the photoreceptor mutants in response to HL may reflect their lower ability to withstand stress than the wild type. Melatonin treatment promoted the downregulation of *APX2* in the WT treatment under HL as a result of decreased photodamage but contributed to a further four-fold increase in the expression of *phyA/B.* A slight, although insignificant, increase in expression was also noted for *cry1/2*. These results show that, upon MT + HL treatment, the mutants seem to encounter more oxidative stress and attempt to cope with it through further elevation in the expression of *APX* (Figure 4).

### 3.3. Exogenous MT Reduced the Photosynthetic Activity and Expression of Photosynthesis-Related Genes in cry1/2 and phyA/B Under HL Stress

The increased susceptibility of the mutants to excess light in the presence of MT was also inferred from their photosynthetic activity data. Compared with those of the control, the ratios of variable fluorescence to maximum fluorescence (Fv/Fm) and the effective quantum yield of PSII (Φ_PSII_) measured immediately after HL treatment strongly decreased in *phyA/B*, indicating dramatic photoinhibition (Table 1). Furthermore, neither parameter exceeded the stress values under combined treatment with stress and melatonin. In contrast, the application of melatonin to LER under HL increased the Fv/Fm ratio as well as Φ_PSII,_ providing efficient protection against photooxidative stress. Similar trends for these indices were noted for *cry1/2* indicating partially different responses of mutants to stress.

Nonphotochemical chlorophyll fluorescence quenching (NPQ), which is considered to be a major factor in the rapid regulation of light harvesting and protection of PSII reaction centers against photodamage [29], increased under excessive light in the WT and in *phyA/B*. However, following HL + MT application, the value of NPQ decreased to control levels in the WT but increased even more in *phyA/B.* Thus, it appears that the mutant was more vulnerable to HL in the presence of MT. In *cry1/2*, the impacts of HL or HL + MT on NPQ were completely different. The initial values decreased significantly under stress or MT treatment, especially under the combined HL + MT treatment. Hence, *cry1/2* suffered even more than *phyA/B* from both HL and MT, indicating that its photosynthetic machinery was undergoing a process of degradation. In summary, the application of MT at least in part could not reverse the deleterious effects of excessive irradiation on the mutants or improve their photosynthetic capacity.

**Figure 5:**
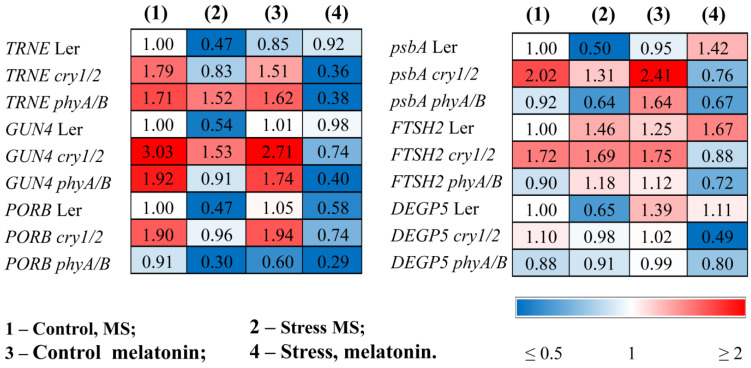
Effects of HL stress and melatonin on photosynthesis-related gene expression wild-type and mutant plants were grown on Murashige and Skoog media in Petri dishes for two weeks under a photoperiod of 16 h in the light/8 h in the dark at 23 °C and 60 μmol m^−2^ s^−1^ (control). Melatonin at a concentration of 50 μm was used for treatment. The experimental plants were exposed to light stress for 24 h at 600 μmol m^−2^ s^−1^ (stress). The numbers indicate the baseline ratio of the expression of each gene in the wild type and mutants without treatments and their values under experimental conditions. Statistical analysis for this figure is presented in Table S2.

The changes in photosynthetic activity in the mutants treated with MT + HL may be due to the altered content of photosynthetic pigments and the modified expression of genes involved in the chlorophyll biosynthesis pathway. The total carotenoid and chlorophyll (*a* + *b*) contents, which decreased under stress conditions, were partly recovered in the LER following the combined application of HL and melatonin (Table 1). However, these indicators remained at stress-reduced levels in melatonin-treated mutants. The reduced expression of *trnE*, *GUN4*, and *PORB*, encoding key steps of chlorophyll biosynthesis under HL stress, was also partially restored by combined MT + HL treatment in WT plants or remained unchanged, but the effect of melatonin treatment was not apparent in stressed mutants. Moreover, melatonin even contributed to the further downregulation of these genes (Figure 5, Table S2).

In parallel, HL caused a decrease in the accumulation of transcripts of the *psbA* gene encoding the PSII protein D1. Exogenous melatonin maintained the expression of this gene at a relatively high level in the stressed wild type, mitigating photodamage, but had no effect or even contributed to its further downregulation in *cry1/2* and *phyA/B* (Figure 5).

The lower photochemical efficiency may be the consequence of a hampered repair cycle of PSII components. Therefore, we next analyzed the expression of the *FTSH2* and *DEGP5* encoding proteases involved in the D1 repair cycle through proteolysis of the damaged D1 proteins and concurrent de novo synthesis [29]. The expression of *FTSH2*, encoding one of the major isoforms of ATP-dependent FTSH metalloproteases, was upregulated by stress and increased further when wild-type samples were treated with MT + HL, thus demonstrating the proper degradation of damaged D1, which is necessary for protection from photoinhibition (Figure 5). Additionally, the transcripts of the ATP-independent DEG endopeptidase DEGP5, whose expression was strongly reduced under HL in the WT, were effectively restored in the MT + HL-treated samples. Conversely, basic steady-state mRNA levels of *DEGP5*, as well as of *FTSH2* transcripts, were either upregulated or did not change in the mutants under HL but were downregulated under combined treatment with MT and HL, especially in *cry1/2.* These findings indicate that melatonin treatment differentially regulates the expression of genes encoding proteases involved in the D1 repair cycle in WT and photoreceptor mutants and may, at least in part, account for the impaired recovery of PSII proteins and decreased photosynthetic activity.

## 4. Discussion

It is generally accepted that melatonin acts as an effective antioxidant to protect organisms from oxidative stress. However, we revealed a paradoxical role of melatonin in the response of *Arabidopsis* photoreceptor mutants to high light stress. Disruption of the phytochrome and cryptochrome genes in the *cry1/2* and *phyA/B* mutants contributed to reduced tolerance to excessive radiation when coupled with melatonin treatment. This claim is based on increased TBAR levels and electrolyte leakage as well as a decrease in photosynthetic efficiency compared with those of plants stressed by HL in the absence of melatonin. In addition, we assessed the expression of key markers of the HL response, which also failed to exhibit increased stress tolerance in the mutants.

Moreover, the parameters of *Landsberg erecta*, which is a parental ecotype of the mutants, improved following melatonin application under HL, in accordance with the long-standing view that the antioxidant properties of melatonin are able to fortify plants subjected to abiotic stress [30]. Efficient scavenging of ROS is considered to be the main molecular mechanism by which melatonin mitigates stress damage. In particular, melatonin-mediated HL tolerance in *Arabidopsis thaliana* has been largely attributed to its ability to scavenge ROS either directly or indirectly through the induction of ROS-responsive antioxidant genes [9,10].

The endogenous MT content is believed to be an important determinant of the plant’s response to stress. Melatonin overproduction in transgenic plants improved stress tolerance in response to salt, cadmium, heat, drought, herbicides, ultraviolet-B light, and pathogens [31]. In our study, the basal levels of MT were lower in both mutants than in the WT, but after joint MT + HL treatment, they exceeded the WT values by approximately two-fold. However, the expression of MT synthesis genes, whose expression was elevated in untreated mutants, decreased following MT application under high irradiance. Similar results were recently reported by Hwang and Back [32]. They showed that the suppression of cryptochrome1b in rice reduced the melatonin content but increased the expression of the melatonin biosynthesis genes *T5H*, *SNAT1*, *SNAT2*, and *COMT*, suggesting that feedback regulation compensates for low melatonin levels. Accordingly, in our study, a decrease in the steady-state MT level in the mutants promoted the upregulation of MT biosynthesis genes, and an increase in the MT content under stress conditions caused the downregulation of MT synthesis genes.

Elevated melatonin production plays a pivotal role in preventing damage from various stresses. Instead, the increase in melatonin levels in the mutants under combined excessive radiation and MT application contributed to a deterioration in stress resistance despite similar levels of total antioxidant capacity in HL- or HL + MT-treated seedlings. This clearly contradicts the idea of an indisputable protective role for melatonin which is associated with scavenging excess ROS, stimulating the activity of antioxidant enzymes, suppressing pro-oxidant enzymes, and minimizing lipid peroxidation [33,34].

In an attempt to clarify the physiological relevance of this discrepancy, we compared ROS production in WT and the mutants, since the melatonin-mediated decrease in ROS production may contribute to an unexpected outcome and perturb the balance in ROS production, which should be above the cytostatic level but below the cytotoxic level [18]. ROS in higher plants regulate development, differentiation, redox levels, stress signaling, interactions with other organisms, systemic responses, and cell death and are therefore essential for the maintenance of normal physiological and metabolic functions. On the other hand, ROS are toxic byproducts of aerobic metabolism capable of inducing DNA, RNA, protein, and membrane oxidation and damage.

In our experiments, the ROS levels decreased markedly following the joint application of MT + HL to the WT plants, whereas the *cry1/2* seedlings accumulated more ROS than the stress-induced plants. In *phyA/B*, the ROS levels were substantially lower than those in WT or cry1/2, which is consistent with the positive role of PhyB in ROS production during excess light stress [17]. Furthermore, the ROS levels did not change following the HL + MT treatment compared with the elevated values under excess light (H_2_O_2_) or even increased (O_2_^−^). Hence, the high capacity of melatonin to detoxify ROS under HL stress is mediated by photoreceptors. However, it is worth noting that our results for the *phyA/B* mutant contradict, to a certain extent, the findings of Fichman et al. [17], according to which H_2_O_2_ accumulates in WT but not *phyB* in response to stress. This discrepancy is possibly explained by the difference in stress duration (24 h vs. 50 min) affecting photooxidative stress tolerance. Additionally, it should be noted that ROS-dependent reactions of *phyA/B* and *cry1/2* to stress and melatonin were somewhat different, combining greater vulnerability of some parameters and lesser vulnerability of others. However, despite the specificity of integration of stress-induced effects, the overall response of the mutants indicates their greater susceptibility to HL during MT treatment compared to the wild type.

PhyB and Cry1 have been shown to regulate the expression of thousands of transcripts. In particular, RNA-Seq analysis of *phyB* mutants under excess white light treatment revealed a large number of differentially expressed genes involved in high light, wounding, drought, heat, and salt responses. In addition, they contained a high number of altered H_2_O_2_−, brassinosteroid-, and jasmonic acid-responsive transcripts [17]. On the other hand, Cry1 has been shown to define a range of light-dependent plant responses, including de-etiolation, photoentrainment of the circadian clock, phototropic curvature, and chloroplast relocation [35,36]. Furthermore, cryptochromes also act as key regulators of a range of plant stress responses, such as drought, salinity, heat, and high radiation [37]. According to genome-wide gene expression data, the dominant groups of genes that were misregulated in *cry1* under high irradiance encode enzymes involved in the phenylpropanoid pathway, components of transcriptional regulation, and proteins associated with stress responses, in addition to proteins with unknown function [13].

The light-sensitive phenotype of photoreceptor mutants under MT treatment could be explained by the misregulation of stress-related genes, which are targets for melatonin action. Transcriptome analysis of *Arabidopsis* plants revealed that almost 40% of the genes whose expression differed in response to the external application of melatonin, including those encoding stress receptors, kinases, transcription factors, and downstream genes encoding end products directly used for stress defense, were related to plant stress defense. Furthermore, the expression of many cell wall-associated genes and genes involved in redox pathways is significantly altered by melatonin treatment [4]. At least some of these genes were misregulated in the photoreceptor mutants.

In particular, the expression of the stress-related genes *EXPA8 (At2g40610)*, encoding expansin A8, or *KFB20 (At1g80440)*, which negatively regulates phenylpropanoid biosynthesis, is strongly dysregulated in *cry1* and significantly affected by 1 mM melatonin [4,13]. According to our unpublished data, 10 of 77 genes misregulated in the *cry1* mutant under HL stress were MT responsive in Col0. One such MT-regulated gene, *HBI1* (basic helix-loop-helix (bHLH) DNA-binding superfamily protein), acts as a new CRY1-interacting protein. The signaling mechanism of CRY1 involves direct blue light-dependent interaction with the transcription factor HBI1 to regulate its transcriptional activity and photomorphogenesis [38].

Concomitantly, MT treatment altered the expression of a set of phyB-dependent genes, such as *AT2G45220* and *AT3G45970* [4,17], which encode cell wall-related genes, and *BZIP34 (AT2G42380)* and *AT5G25930*, which are involved in signal transduction. We speculate that photoreceptors act upstream of MT-dependent genes, modulating their expression and ensuring the protective functions of MTs. In particular, photoreceptors can interact via phytochrome-interacting factors (e.g., PIF4) with DNA-binding fragments present in the promoter regions of MT-related genes, which act as transcriptional activators or repressors.

Links between photoreceptors and melatonin may not be limited to phytochromes and cryptochromes but seem to be shared by other photoreceptors, such as phototropins, Zeitlupe family proteins, and UVR8, given their involvement in light stress responses [39]. How precisely photoreceptors induce the reprogramming of MT target genes remains to be elucidated. Future genome-wide studies of transcriptional regulation may provide the basis for deciphering the molecular mechanisms linking MT and photoreceptor-mediated responses to high radiation intensity. The paradoxical impact of melatonin in photoreceptor mutants suggests that its multifaceted role under stress conditions may depend on a specific genetic background. Like ROS, which have traditionally been referred to as the double-edged sword of life [18], melatonin should be treated as a plant weapon system that combines beneficial and toxic effects.

## 5. Conclusions

Since its discovery in 1995, phytomelatonin has earned a reputation as a profound biostimulating molecule in plants involved in adaptation to ever-changing and frequently adverse environmental conditions. However, we found a paradoxical role of melatonin in the response of *Arabidopsis* photoreceptor mutants to severe light stress, which is inconsistent with its role as an antioxidant and signaling molecule capable of reducing ROS production and regulating the expression of ROS-related genes. The increase in melatonin levels in *cry1/2* and *phyA/B* mutants under combined exposure to HL stress and MT application contributed to a deterioration in stress resistance. In contrast to the parental form of *Landsberg erecta*, the mutants presented elevated TBAR levels and electrolyte leakage, as well as reduced photosynthetic efficiency, compared with the HL values in the absence of melatonin. The reduced stress resistance of the mutants was also confirmed by analysis of the transcript abundance of ROS markers and enzymatic scavengers. Because a melatonin-mediated decrease in ROS levels may contribute to an imbalance in ROS production below the levels required for basic biological processes, we assessed the impact of MT on ROS removal. Unexpectedly, MT treatment and increased endogenous levels of MT promoted increased ROS levels in *cry 1/2* and *phyA/B*. This discrepancy could be explained by misregulation of melatonin target genes involved in stress responses in the mutants. We therefore hypothesize that the role of melatonin in enhancing HL stress tolerance is mediated via photoreceptors acting upstream of MT-regulated genes. However, further research is needed to pinpoint key modules involved in photoreceptor-driven MT responses.

## Figures and Tables

**Table 1:** Effects of HL stress and melatonin on the photosynthetic pigment content and chlorophyll fluorescence parameters. The data presented in the table are mean values (*n* ≥ 3). Error bars represent measurement errors. Different letters indicate statistically significant differences between variants within the same genotype at *p* < 0.05 (ANOVA with Tukey’s post hoc multiple comparison test). Asterisks indicate statistically significant differences between the mutants and the wild type under the corresponding treatment type at *p* < 0.05 (Student’s *t* test).

Genotype	Control/MS	Stress/MS	Control/Melatonin	Stress/Melatonin
Fv/Fm				
Ler	0.821 ± 0.011 ^a^	0.550 ± 0.027 ^c^	0.826 ± 0.014 ^a^	0.661 ± 0.019 ^b^
*cry1/2*	0.819 ± 0.009 ^a^	0.541 ± 0.030 ^c^	0.806 ± 0.016 ^a^	0.645 ± 0.025 ^b^
*phyA/B*	0.814 ± 0.013 ^a^	0.599 ± 0.011 ^b^	0.808 ± 0.021 ^a^	0.593 ± 0.029 ^b^
Φ_PSII_				
Ler	0.732 ± 0.019 ^a^	0.488 ± 0.016 ^c^	0.709 ± 0.010 ^a^	0.579 ± 0.010 ^b^
*cry1/2*	0.704 ± 0.015 ^a^	0.473 ± 0.018 ^c^	0.692 ± 0.017 ^a^	0.572 ± 0.022 ^b^
*phyA/B*	0.678 ± 0.019 ^a^	0.435 ± 0.024 ^b^	0.671 ± 0.018 ^a^	0.456 ± 0.029 ^b^
NPQ				
Ler	0.187 ± 0.006 ^c^	0.337 ± 0.015 ^a^	0.186 ± 0.011 ^c^	0.228 ± 0.018 ^b^
*cry1/2*	0.480 ± 0.021 ^a^*	0.395 ± 0.032 ^a^	0.391 ± 0.027 ^a^	0.360 ± 0.022 ^a^
*phyA/B*	0.326 ± 0.016 ^c^	0.484 ± 0.019 ^b^	0.313 ± 0.013 ^c^	0.691 ± 0.020 ^a^
Chlorophyll content (*a + b*), mg g^−1^ (FM)				
Ler	1.107 ± 0.051 ^a^	0.369 ± 0.019 ^b^	1.052 ± 0.039 ^a^	0.733 ± 0.026 ^c^
*cry1/2*	0.931 ± 0.034 ^a^*	0.632 ± 0.037 ^c^	0.821 ± 0.029 ^b^	0.643 ± 0.046 ^c^
*phyA/B*	0.816 ± 0.043 ^a^*	0.452 ± 0.027 ^b^	0.746 ± 0.039 ^a^	0.437 ± 0.022 ^b^
Carotenoid content, mg g^−1^ (FM)				
Ler	0.186 ± 0.009 ^a^	0.149 ± 0.007 ^b^	0.178 ± 0.011 ^a^	0.180 ± 0.013 ^a^
*cry1/2*	0.189 ± 0.014 ^a^	0.151 ± 0.009 ^bc^	0.169 ± 0.011 ^ab^	0.132 ± 0.009 ^c^
*phyA/B*	0.158 ± 0.012 ^a^	0.108 ± 0.009 ^b^	0.130 ± 0.010 ^a^	0.109 ± 0.011 ^b^

## Data Availability

Data are contained within the article and Supplementary Materials.

## Supplementary Materials

The following supporting information can be downloaded from: https://www.mdpi.com/article/10.3390/antiox14040458/s1, Table S1. List of primers used for RT-qPCR. Table S2. Effect of stress and melatonin treatment on the relative expression levels of genes.
