# Supplementary material for: Photoreceptors Are Involved in Antioxidant Effects of Melatonin Under High Light in Arabidopsis
Source: Antioxidants (Basel). 2025 Apr 12;14(4):458. doi: 10.3390/antiox14040458 (PMC12023978; doi:10.3390/antiox14040458)
Supplement: Supplementary file 1 [file antioxidants-14-00458-s001.zip › antioxidants-3542972-supplementary.pdf]

Photoreceptors are Involved in Antioxidant Effects of Melatonin under High Light in *Arabidopsis*

Ivan Bychkov†, Anastasia Doroshenko†, Natalia Kudryakova*, and Victor Kusnetsov
К.A. Timiryazev Institute of Plant Physiology RAS, 35 Botanicheskaya St., Moscow, 127276, Russia
*Author to whom correspondence should be addressed.
†These authors contributed equally to this work.

Table S1. List of primers used for RT-qPCR.

Gene name	Locus	Forward primer (5′->3′)	Reverse primer (5′->3′)
SNAT1	At1g32070	TAC GCA ACT TGT GGA ACC ACC T	ATC ATA AAC ATC AAT CTC ACC ACC A
ASMT	At4g35160	GCAAAGAAGCGGTCCC TCCAA	GTCCGTTCTTTGCCT GTGCTTGT
COMT	At5g54160	GGA GTG ACG AAC ATT GCG T	TCT CGG TTC GTT CTT TGC CT
CRY1	At4g08920	GAA CCA ACC AGA CTC AAC CCA AA	ACC TCT GCT CTG CTA TCT CCT ACT GA
CRY2	At1g04400	TGG AAC TCG GAA CAA ACT ATG CG	TAG AAG ACA CAG ATG GGC AAA GAC C
PHYA	At1g09570	GGC AAT CAT CCA AAA TCC AAA CC	ATC ACT TCC TCT CGC TTC AAA CC
PHYB	At2g18790	CAT AGC AGC AGG TGG ACA AGC	CCA GAA TGA TGA GGA AAT AGG ACC
OXI1	At3g25250	CGA CGA CGC TAA ATT GCT TGC T	CAC CGT GAA GAG ACG GAA AGA GA
At3g01830	At3g01830	CTG CTT TGA CAA GAG CCA CCA AG	CAA CTA CTG CAC GTT TGC CAG ACT T
ELIP2	At4g14690	ACG GGA GAC TAG CAA TGG TT	CCT AGA AAC CAC CCG ACA CC
SIG5	At5g24120	AGA TGT TGA TGG TGT TGG AGC	GAC TCT CTT TCG GCT TCA ATG
AOX1a	At3g22370	GAT TGG AGG TAT GAG ATT CGC	CGG TGG ATT CGT TCT CTG TTT
APX2	At3g09640	CTA AGA GAT GTG TTT GGT CGG ATG G	GAA TCC TGA ACG CTC CTT GTG G
TRNE	ArthCt097	CCC AGGM GGA AGT CGA ATC C	GCC CCC ATC GTC TAG TGG TTC
GUN4	At3g59400	CTG CTC TGC TTC TTC CAC CTC CT	GTG TCT CCT CGT CGG CTT GTC T
PORB	At4g27440	GCT ATG ATT GAT GGA GGA GAT TTC G	AAG AGG GCA CGG AAG AGA GGA A
PSBA	Atcg00020	CGG TGC CAT TAT TCC TAC TTC T	CTT GCC CGA ATC TGT AAC CT

FTSH2	At2g30950	CTT CGG CAC AAA GCG ATG TCA	CCT GAA TTC GTC ACC GCC AA
DEGP5	At4g18370	ATG ACC ATG GCT CTT GCT TCT TCT	AGA GCC AAA CTC GAG CCA AAG AT
UBQ10	At4g05320	GCG TCT TCG TGG TGG TTT CTA A	GAA AGA GAT AAC AGG AAC GGA AAC A

Sequence data from this article can be found in the National Center for Biotechnology Information https://www.ncbi.nlm.nih.gov/

Table S2. Effect of stress and melatonin treatment on the relative expression levels of genes. WT and mutant plants were grown on Murashige and Skoog medium in Petri dishes for two weeks under a 16 h light / 8 h dark photoperiod at 23°C and 60 µmol m^{-2} s^{-1}. Melatonin at a concentration of 50 µm was used for treatment. Experimental plants were exposed to high light stress for 24 h at 600 µmol m^{-2} s^{-1}. Control plants were kept at a light intensity of 60 µmol m^{-2} s^{-1}. The data presented in the table are the mean values ($n \geq 3$). Error bars represent SEs. Different letters denote statistically significant differences between variants within the same genotype at $p < 0.05$ (ANOVA with post hoc Tukey's multiple-comparison test); asterisks indicate statistically significant differences between the control variants of the mutants and the wild type at $p < 0.05$ (t test).

**Plants**	**Control, MS**	**Stress, MS**	**Control, melatonin**	**Stress, melatonin**
CRY1				
**WT**	1.000±0.116^{ab}	0.408±0.055^{c}	1.352±0.138^{a}	0.853±0.091^{b}
cry1/2	-	-	-	-
phyA/B	1.091±0.177^{a}	0.482±0.101^{b}	1.102±0.155^{a}	0.453±0.091^{b}
CRY2				
**WT**	1.000±0.147^{a}	0.272±0.023^{b}	1.134±0.120^{a}	0.293±0.509^{b}
cry1/2	-	-	-	-
phyA/B	1.219±0.145^{a}	0.709±0.121^{b}	1.538±0.191^{a}	0.737±0.126^{b}
PHYA				
**WT**	1.000±0.130^{a}	0.350±0.027^{c}	1.202±0.125^{a}	0.656±0.071^{b}
cry1/2	4.039±0.582^{a*}	1.666±0.171^{b}	4.225±0.533^{a}	1.224±0.138^{c}
phyA/B	-	-	-	-
PHYB				
**WT**	1.000±0.109^{c}	1.511±0.128^{b}	1.232±0.126^{bc}	2.452±0.308^{a}
cry1/2	1.966±0.238^{b*}	2.610±0.381^{a}	2.114±0.237^{b}	1.513±0.081^{c}
phyA/B	-	-	-	-
OXI1				
**WT**	1.000±0.104^{d}	8.113±0.108^{a}	2.152±0.220^{c}	5.848±0.079^{b}
cry1/2	0.683±0.074^{d*}	6.113±0.708^{b}	0.952±0.101^{c}	9.348±0.981^{a}
phyA/B	0.951±0.086^{b}	8.761±0.775^{a}	0.925±0.081^{b}	11.373±1.040^{a}
AT3G01830				
**WT**	1.000±0.111^{c}	2.109±0.222^{a}	1.451±0.127^{b}	1.848±0.163^{ab}
cry1/2	0.288±0.043^{c*}	7.104±0.812^{b}	0.209±0.019^{c}	19.852±1.993^{a}
phyA/B	0.512±0.051^{b*}	4.894±0.506^{a}	0.253±0.033^{c}	6.096±0.708^{a}
ELIP2				
**WT**	1.000±0.077^{c}	36.226±4.108^{a}	0.9152±0.083^{c}	11.848±1.236^{b}

cry1/2	1.012±0.094[c]	2.263±0.255[b]	1.217±0.118[c]	5.420±0.637[a]
phyA/B	1.197±0.082[c]	10.555±1.813[b]	1.279±0.133[c]	20.674±3.130[a]
SIG5				
**WT**	1.000±0.126[b]	6.201±0.671[a]	0.682±0.053[c]	1.152±0.121[b]
cry1/2	0.904±0.107[a]	0.855±0.074[a]	0.802±0.067[a]	0.987±0.095[a]
phyA/B	1.321±0.120[b]	2.301±0.199[a]	1.259±0.081[b]	2.656±0.321[a]
AOX1a				
**WT**	1.000±0.104[a]	2.116±0.108[a]	1.092±0.063[a]	1.046±0.088[a]
cry1/2	1.044±0.109[b]	2.203±0.215[a]	1.107±0.106[b]	2.370±0.301[a]
phyA/B	1.053±0.116[a]	4.230±0.447[a]	1.307±0.144[b]	4.336±0.501[c]
APX2				
**WT**	1.000±0.088[c]	19.403±1.006[a]	1.342±0.120[c]	9.829±0.601[b]
cry1/2	0.517±0.048[c*]	3.061±0.262[a]	1.007±0.091[b]	4.421±0.506[a]
phyA/B	1.204±0.130[c]	5.113±0.253[b]	1.121±0.113[c]	20.713±3.005[a]
MAPK6				
**WT**	1.000±0.100[a]	1.313±0.108[a]	1.415±0.124[a]	1.365±0.145[a]
cry1/2	1.517±0.201[a*]	1.006±0.088[b]	1.539±0.177[a]	1.023±0.092[b]
phyA/B	0.914±0.084[b]	1.202±0.290[ab]	1.527±0.101[a]	1.017±0.154[ab]
TRNE				
**WT**	1.000±0.059[a]	0.469±0.036[b]	0.852±0.076[a]	0.920±0.088[a]
cry1/2	1.799±0.202[a*]	0.831±0.079[b]	1.514±0.170[a]	0.362±0.051[c]
phyA/B	1.711±0.193[a*]	1.520±0.391[a]	1.622±0.203[a]	0.376±0.044[b]
GUN4				
**WT**	1.000±0.112[a]	0.542±0.045[b]	1.010±0.104[a]	0.978±0.092[a]
cry1/2	3.032±0.284[a*]	1.531±0.126[b]	2.713±0.366[a]	0.741±0.083[c]
phyA/B	1.917±0.284[a*]	0.913±0.090[b]	1.736±0.266[a]	0.402±0.051[c]
PORB				
**WT**	1.000±0.096[a]	0.473±0.039[b]	1.050±0.116[a]	0.582±0.051[b]
cry1/2	1.913±0.202[a*]	0.965±0.107[b]	1.937±0.224[a]	0.739±0.079[b]
phyA/B	0.906±0.088[a]	0.298±0.027[c]	0.604±0.055[b]	0.295±0.032[c]
LHCB2				
**WT**	1.000±0.094[a]	0.113±0.015[c]	0.259±0.120[b]	0.110±0.011[c]
cry1/2	1.811±0.193[a*]	0.188±0.022[b]	1.613±0.169[a]	0.131±0.014[c]
phyA/B	3.002±0.337[a*]	0.407±0.051[b]	3.805±0.420[a]	0.350±0.032[b]
psbA				
**WT**	1.000±0.104[a]	0.502±0.047[b]	0.950±0.091[a]	1.415±0.154[a]
cry1/2	2.016±0.205[a*]	1.307±0.140[b]	2.410±0.251[a]	0.755±0.078[c]
phyA/B	0.917±0.086[b]	0.636±0.072[c]	1.635±0.188[a]	0.674±0.076[c]
FTSH2				
**WT**	1.000±0.101[b]	1.456±0.132[a]	1.252±0.120[ab]	1.672±0.079[a]
cry1/2	1.721±0.163[a*]	1.684±0.156[a]	1.746±0.191[a]	0.880±0.091[b]
phyA/B	0.900±0.072[ab]	1.182±0.125[a]	1.116±0.118[a]	0.724±0.066[b]
DEGP5				
**WT**	1.000±0.095[a]	0.652±0.061[b]	1.398±0.145[a]	1.113±0.119[a]
cry1/2	1.103±0.098[a]	0.983±0.094[a]	1.023±0.106[a]	0.496±0.052[b]
phyA/B	0.887±0.084[a]	0.912±0.084[a]	0.995±0.106[a]	0.802±0.075[a]
